# “The Great Masquerader”: Sonographic Pictorial Review of Testicular Tuberculosis and its Mimics

**DOI:** 10.25259/JCIS-14-2019

**Published:** 2019-06-14

**Authors:** Pankaj Nepal, Vijayanadh Ojili, Swachchhanda Songmen, Neeraj Kaur, Thomas Olsavsky, Arpit Nagar

**Affiliations:** 1 Departments of Radiology, St. Vincent’s Medical Center, Bridgeport, CT, USA; 2 Departments of Radiology, University of Texas Health, San Antonio, TX, USA; 3 Departments of Radiology, Ohio State University Wexner Medical Center, Columbus, OH, USA.

**Keywords:** Ancillary imaging, Mimics, Testicular tuberculosis, Ultrasound

## Abstract

Testicular tuberculosis (TB) is an uncommon presentation of extrapulmonary TB. Although rare in incidence, it is a great masquerader and should be kept in consideration while assessing focal abnormalities involving the testis. Ultrasound findings alone may be non-specific and mimic other diagnoses including infection, inflammation, tumor, infarct, and trauma. The main objective of this sonographic pictorial review is to discuss the imaging findings, specific differentiating features against each differential and use of ancillary imaging findings whenever available. Concurrent involvement of epididymis, septated hydrocele, scrotal wall edema, and calcification of tunica vaginalis provides strong evidence in an appropriate setting. Available extratesticular ancillary imaging findings must be correlated for correct diagnosis due to non-specific imaging and clinical presentation. Misdiagnosis of scrotal TB may lead to otherwise avoidable epididymo-orchiectomy.

## INTRODUCTION

Genitourinary tuberculosis (TB) is the most common form of extrapulmonary TB, representing up to 20% of cases.^[1]^ However, testicular TB is rare and represents only up to 3% of the cases of genitourinary TB.^[1,2]^ Genitourinary TB may occur at any age, but more common between the third and fifth decade of life.^[3]^ Concurrent pulmonary and renal TB is seen in 50% and 80–85% of the cases of genitourinary TB.^[4]^ Incidence of extrapulmonary TB is increasing not only in endemic regions but also all across the world due to immigration, immunosuppression, and HIV infection. Imaging features of testicular TB are non- specific and often impossible to distinguish from other more common pathologies such as tumor, infection, inflammation, and infarction. Testicular imaging is limited to the use of magnetic resonance imaging (MRI) and ultrasound. Computed tomography (CT) scan is not reliable in diagnosing and characterizing testicular pathology. Ancillary tests such as positive chest radiograph or tuberculin test support the diagnosis. However, negative results cannot reliably exclude the diagnosis.^[2,3]^ Unfortunately, only half of the patients have evidence of active TB at presentation.^[2]^ Antitubercular medicines are the mainstay of treatment; however, orchiectomy may be required for both diagnosis and treatment. Differential diagnoses of testicular TB are summarized in this article with specific teaching points that may help in differentiating with its close mimics.

## DISCUSSION

**Figure 1:**
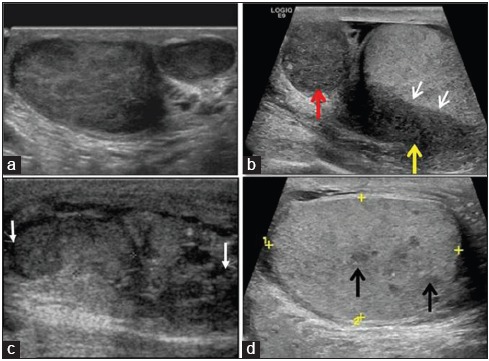
Various examples of gray scale sonographic patterns of testicular tuberculosis. (a) Diffusely enlarged right testis with heterogeneous hypoechoic pattern, (b) diffusely enlarged head (red arrow) and body (yellow arrow) of epididymis with infiltration of adjacent testes parenchyma (white arrows) showing homogenous hypoechoic pattern, (c) nodular enlarged heterogeneously hypoechoic testes, with ill-defined nodules (small white arrows) virtually indistinguishable from tumor, (d) ill-defined tiny hypoechoic nodules (black arrows) in testicular parenchyma.

**Figure 2:**
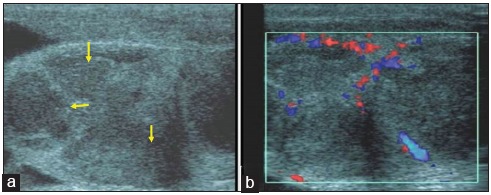
A 44-year-old man with testicular tuberculosis who presented with 6 months history of testicular pain. (a) Gray scale sonographic image demonstrates an enlarged and heterogeneous testis with the presence of multiple ill-defined focal hypoechoic lesions (small yellow arrows). (b) Color Doppler shows increased vascularity along the periphery of the hypoechoic lesions. These imaging findings are non-specific and may be seen in both inflammatory and neoplastic conditions. A diagnosis of testicular tuberculosis was made the following orchiectomy.

**Figure 3:**
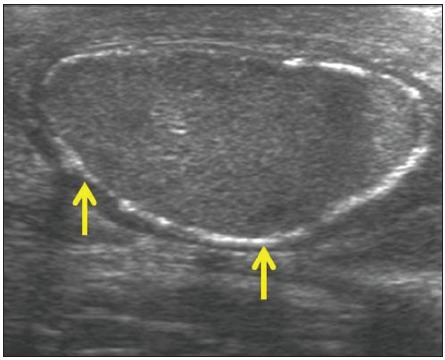
A 42-year-old man with healed testicular tuberculosis: Gray scale sonographic image demonstrates smooth peripheral calcification along the tunica vaginalis (yellow arrows). The patient had a remote history of pulmonary tuberculosis, which was treated with antitubercular drugs.

Ultrasound is the investigation of choice for diagnosis of testicular TB. Various sonographic patterns have been described for testicular TB^[5]^ [Table 1 and Figure 1]. The sonographic appearances of testes can be explained by various pathologic stages of tubercular infection which include caseous necrosis, granulomas, and healing by fibrosis and calcification [Figure 2]. Due to vague sonographic patterns, imaging features are often non-specific and difficult to distinguish TB from other inflammatory causes, tumor, or infarction. The disease may progress to involve entire epididymis and testis or may heal with fibrocalcific changes. Calcification is seen in 10% of cases [Figure 3].^[6,7]^

**Table 1:** Gray scale sonographic patterns of testicular TB.

Diffusely enlarged heterogeneously hypoechoic testis
Diffusely enlarged homogeneously hypoechoic testis
Nodular enlarged heterogeneously hypoechoic testis
Multiple small hypoechoic nodules in the enlarged testis (miliary type)

TB: Tuberculosis

Epididymis is the most common site of tubercular infection in scrotum. Tubercular orchitis usually results from contiguous extension from the epididymis. Isolated orchitis in the absence of epididymal involvement is rare, however, possible with hematogenous spread.^[8,9]^ Tubercular epididymitis occurs first due to early involvement from retrograde spread of mycobacteria through urinary reflux. Again, tail of the epididymis has greater blood supply and might be another reason for early involvement. Concurrent presence of epididymis involvement and testicular lesion favors the diagnosis of infection.^[10]^

Complications of testicular TB include scrotal abscess, scrotal sinus tract, and extratesticular calcification. Intrascrotal extratesticular calcification affects the epididymis and the tunica vaginalis covering the testes. Scrotal fistula formation has a poor prognosis.

### Specific ultrasound imaging features of testicular TB

**Figure 4:**
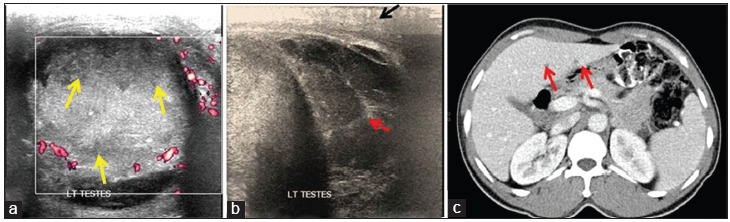
A 43-year-old male with complicated testicular tuberculosis presenting with scrotal pain and swelling. (a) Color Doppler sonographic image demonstrates diffuse, ill-defined heterogeneous hypoechoic area with increased peripheral vascularity but lack of central vascularity (yellow arrows). (b) Gray scale ultrasound image showing scrotal wall edema (black arrow) and multiseptated hydrocele and debris (red arrow). (c) Contrast-enhanced axial computed tomography image of abdomen in same patient shows hypoechoic lesions in the left lobe of liver (red arrows) likely representing tubercular granulomas.

Concurrent involvement of the epididymis along with testes, scrotal wall thickening, and septated or infected hydrocele favors infection rather than tumor [Figure 4]. However the testicular tumors at advanced stages may also involve the epididymis.^[1-4]^ Miliary nodules in testes should alert a radiologist toward tubercular etiology. Smooth peripheral calcification of tunica vaginalis is also a specific feature of testicular TB in contrast to intratesticular coarse calcifications seen in malignancy. Color Doppler sonography is also helpful in differentiating TB from infarcts and tumors. TB orchitis commonly occurs with the involvement of lower urinary tract and sometimes with concurrent renal involvement.^[5]^

On MRI, the tubercular granulomas usually appear hypointense in T2-weighted (T2W) images and show variable contrast enhancement. Rarely, acute involvement may demonstrate T2 hyperintense signal mimicking bacterial orchitis.

### Testicular TB mimics

Imaging appearance of testicular TB is non-specific and masquerades non-specific infection, inflammation, tumor, trauma, and infarct.

### Sarcoidosis

Sarcoidosis is a chronic granulomatous disease with multisystem involvement that can rarely involve the testes. The most common presentation of genitourinary sarcoidosis is epididymitis, which is often bilateral. Most often, the epididymitis is asymptomatic, but patients may present with pain or scrotal mass. In most cases, sarcoidosis is diagnosed before genitourinary involvement occurs. Like TB, isolated involvement of the testis without inflammation of epididymis is very rare. On ultrasound, sarcoid granulomas appear as single or multiple hypoechoic nodules within the testes, mimicking TB.^[11,12]^ The sarcoid granulomas are similar to tubercular granulomas and are hypointense on T2W MRI and usually show contrast enhancement. Management of sarcoidosis is completely different as steroid therapy is beneficial. Multiple bilateral granulomas with simultaneous involvement of the epididymis and testes are indistinguishable from TB; however, in conjunction, other systemic manifestations such as pulmonary involvement should raise the suspicion of sarcoidosis.

**Figure 5:**
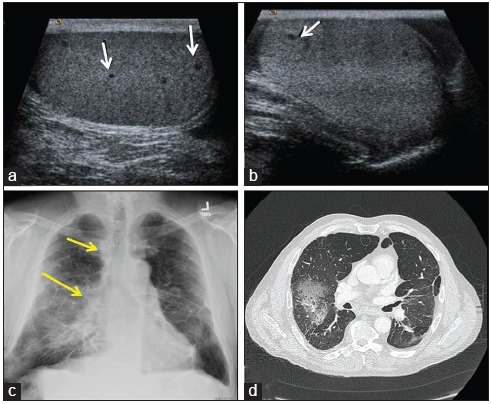
A 55-year-old-male with sarcoidosis who presented with the right testicular pain. (a and b) Gray scale sonographic image of testis revealed several, tiny bilateral hypoechoic lesions (white arrows) on both testes (A-right, B-left testis). (c) Frontal chest radiograph shows the right hilar and paratracheal lymphadenopathy (yellow arrows) and bilateral interstitial and airspace opacities more on the right side. (d) Axial computed tomography chest image on lung window demonstrates asymmetric ground-glass opacities with surrounding pulmonary fibrosis and architecture distortion more prominent on the right.

Similar to testicular TB, in sarcoidosis (a) testicular involvement without epididymitis is rare, (b) granulomas appear hypoechoic on ultrasound, and (c) granulomas show T2 hypointensity and enhancement [Figure 5]. However, concurrent systemic manifestations of sarcoidosis will provide clue to the diagnosis of sarcoidosis.

### Lymphoma

Testicular lymphoma represents about 5% of all testicular tumors. However, it is the most common testicular tumor in elderly men >60 years.^[13,14]^ It is the most common bilateral testicular tumor. Lymphoma can involve the testes either as primary site of extranodal disease or as secondary involvement of systemic disease. Lymphoma in testes is most often secondary and is usually seen in disseminated lymphoma – which makes the diagnosis of the testicular lymphoma easier. Most of them are diffuse large B-cell non-Hodgkin’s lymphoma.

**Figure 6:**
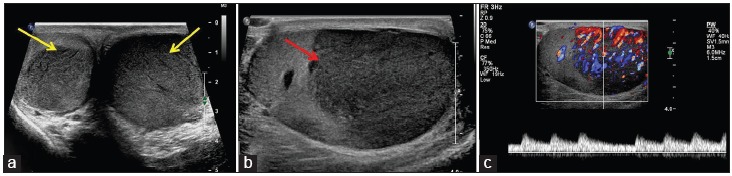
A 70-year-old elderly male with lymphoma who presented with scrotal swelling. (a) Gray scale ultrasound shows bilateral enlarged testes with bilateral hypoechoic masses (yellow arrows). (b) Note the diffuse involvement of the left testis by hypoechoic mass (annotated with red arrow). (c) Color Doppler image showing profound vascularity concerning for tumor. A presumptive diagnosis of testicular tumor was made based on the patients age, clinical presentation, and sonographic findings. Histopathological examination of the left orchiectomy specimen revealed non-Hodgkin’s lymphoma.

Most common imaging feature in ultrasound is diffuse enlargement of testes with large hypoechoic infiltrative area replacing the testes but characteristically maintaining normal testicular shape. Less commonly, it may present as discrete hypoechoic intratesticular mass which may be solitary or multiple. On color Doppler, increased color flow is noted resembling diffuse inflammation, but without pain or tenderness [Figure 6].^[13]^ Imaging features on ultrasound are non-specific and similar to TB and sarcoidosis, epididymis may be involved which appears enlarged and hypoechoic. On MRI, the soft tissue appears hypointense both in T1W and T2W images with subtle enhancement.^[14]^ Testicular lymphoma should be considered in an elderly male with infiltrative hypoechoic mass in enlarged testes yet maintaining the shape of testes or multiple bilateral non-tender testicular masses with increased vascularity on color Doppler. Diagnosis is with orchiectomy, which is also therapeutic.

### Primary testicular tumors

**Figure 7:**
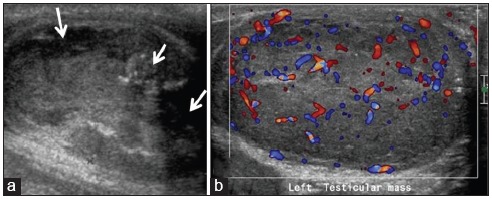
A 30-year-old male with testicular tumor who presented with progressive right scrotal pain and swelling for a month. (a) Gray scale sonographic image demonstrates heterogeneous testis with multiple focal hypoechoic lesions with punctate calcifications (white arrows). The imaging features are simulating malignancy. (b) On color Doppler, peripheral and central vascularity is seen. This was found to be testicular undifferentiated sarcoma.

Testicular tumors are the most common neoplasm in men of the second and third decade of life. Most common presenting symptoms of tumors are scrotal swelling and lump. Testicular tumors and other non-tumorous conditions such as focal infarct, hematoma, and infection have overlapping imaging appearances, i.e., hypoechoic areas with variable color flow [Figure 7]. Thus, a solid testicular mass with internal vascularity should be considered as testicular tumor until proven otherwise. Ultrasound can distinguish between intratesticular versus extratesticular mass; intratesticular mass is more commonly malignant. Seminomatous tumors are more homogenous in contrast to non-seminomatous tumors.

Color Doppler can help differentiate infarction, tumor, and inflammation. On color Doppler, tubercular epididymitis and orchitis demonstrate peripheral vascularity due to granulomas and lack of central flow due to caseation necrosis which is in contrast to tumor which usually shows central vascularity.^[15]^

Concurrent epididymis enlargement with a testicular involvement favors infection rather than tumor because orchitis is preceded by epididymitis.^[16]^ Testicular tumors may also infiltrate the epididymis, but in the advanced stages, when we also expect extratesticular systemic findings. Calcification can also be seen in testicular tumors which are usually intratesticular, coarse, and inhomogeneous distinct from the pattern of calcification in testicular TB. Calcification typical for TB is smooth peripheral involving the tunica.

### Testicular metastasis

Metastasis to the testes is rare. The most common primary that metastasizes to the testicle are carcinoma of prostate, lung, kidney, and colon. Leukemia and metastasis from melanoma can also present in testes. Neuroblastoma, Wilms tumor and sarcoma can metastasize in children. Most often, the metastasis is unilateral but can be bilateral in up to 15% of cases.^[16,17]^ Mean age at presentation is the 5^th^ decade of life which is much older than the age for primary testicular tumor. Testicular metastasis is rare, probably due to low temperature of scrotum where malignant cells find difficult to proliferate as well as due to blood-testicular barrier.^[17]^

### Hematoma and infarcts

**Figure 8:**
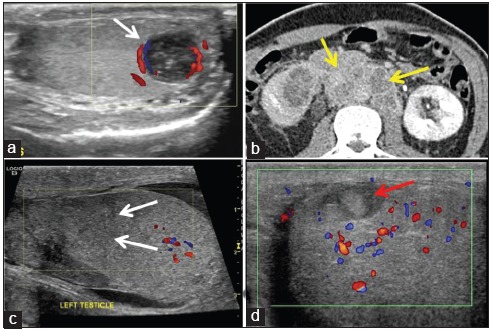
Tuberculosis mimics. A 20-year-old male with scrotal pain. (a) A well-defined heterogeneous hypoechoic lesion in lower pole of testes (white arrow) with peripheral flow on color Doppler. (b) Axial contrast-enhanced computed tomography abdomen of same patient revealed bulky conglomerate lymphadenopathy (yellow arrows) diagnosed as germ cell tumor. (c) A 16-year-old male with trauma. Irregular hypoechoic area in upper pole of testis (white arrows) with minimal hydrocele. (d) A 35-year-old male for follow-up of testicular trauma. Avascular heterogeneous lesion in upper pole of testis (red arrow) diagnosed as hematoma.

Although on imaging testicular hematoma and infarcts simulate testicular TB and tumors, hematoma and infarcts can be differentiated clinically. Testicular infarct presents with acute scrotal pain, color flow is absent or reduced. Area of infarct is avascular and will become more hypoechoic on follow-up. Testicular hematoma is suspected after trauma which appears hypoechoic and shows reduced or absent vascularity on color Doppler. Chronic hematoma may simulate mass, which should be correlated with the history and color Doppler findings [Figure 8]. In uncertain cases, if it seems as an incidental finding associated with trauma, short-term follow-up imaging in 2–4 weeks may be helpful because hematoma will regress in size with time.^[18]^

### Other infectious orchitis

Bacterial orchitis may have similar findings on ultrasound; however, the clinical presentation is different and presents with fever, acute scrotal pain, urinary tract infection, and leukocytosis. On color Doppler, pyogenic orchitis demonstrates markedly increased vascularity opposed to spotty peripheral flow along the tubercular abscess.^[19]^ Progression of abscess to fibrosis or calcification is favorable in tubercular etiology. On MRI, tubercular orchitis usually demonstrates T2 hypointense signal in contrast to pyogenic orchitis which shows hyperintense signal on T2W imaging.

### Adrenal rests

Testicular adrenal rest tumors are benign masses that are found in patients with congenital adrenal hyperplasia with the prevalence of 94%. Adrenal rest tumors are present in childhood; thus, baseline ultrasound screening is recommended to preserve testicular function. Adrenal rest tumors are typically bilateral and located near the mediastinum of testes. Most often, the lesions appear hypoechoic with well-defined margins and demonstrate vascularity on color Doppler.^[20]^

### Importance of extratesticular ancillary findings

**Figure 9:**
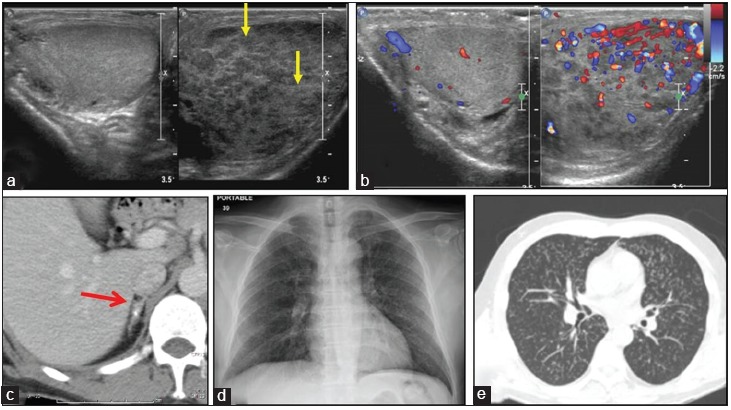
Testicular tuberculosis mimicking tumor; importance of extratesticular ancillary findings. (a) Gray scale sonographic image of scrotum showing enlarged left testis with multifocal ill-defined hypoechoic nodularity (yellow arrows). (b) Color Doppler shows increased vascularity. The left radical orchiectomy revealed caseous granulomatous orchitis and epididymitis. (c) Retrospective analysis of contrast-enhanced computed tomography (CT) abdomen shows calcified right adrenal gland (red arrow). (d) Chest X-ray was normal and (e) CT chest revealed bilateral lung parenchyma studded with multiple miliary tubercles.

It is important to correlate the testicular ultrasound findings with extratesticular ancillary findings. From above discussion, it is clear that testicular TB mimics a wide range of differential diagnosis. Extratesticular findings can guide us to the correct diagnosis [Figure 9].

Both TB and sarcoidosis are granulomatous diseases with overlapping imaging findings. Hilar and mediastinal lymphadenopathy with occasional calcification of affected nodes may be seen in both TB and sarcoidosis. Interstitial lung disease with characteristic thickening of bronchovascular bundles in high-resolution CT favors sarcoidosis. On the other hand, cavity formation favors TB which is seen in <3% of the cases of sarcoidosis. TB must be suspected in patients with testicular lesions and concurrent lower urinary tract symptoms such as voiding difficulty and hematuria. Multiple nodules in testes can be confused with sarcoidosis, but systemic evaluation in difficult cases is useful. Testicular TB is common in the 3^rd^–4^th^ decade of life.^[15]^ Half of the cases of testicular TB will have active disease presentation, thus systemic evaluation and correlation can clinch the diagnosis.

In the elderly age group, diagnostic dilemma develops between testicular malignancy and testicular TB. After the sixth decade, malignancy such as lymphoma is more common. Testicular malignancy may present with bulky retroperitoneal lymphadenopathy which could be important extratesticular ancillary finding. TB can also involve abdominal lymph nodes which are necrotic, hypoechoic/attenuating, calcified with mesenteric predominance. It is also important to search for evidence of trauma to differentiate chronic hematoma from testicular pathology.

## CONCLUSION

Testicular TB is a great masquerader and may mimic a wide range of infectious, inflammatory, and neoplastic processes. Although the sonographic findings of testicular TB are overlapping, knowledge of extratesticular ancillary findings and history is useful in most cases. It is important to accurately diagnose testicular TB and differentiate it from other differentials, especially testicular malignancy as the management is totally different.
